# Shoot Nutrient Content and Nutrient Resorption of *Leymus chinensis* in Various Legume Mixtures

**DOI:** 10.3389/fpls.2018.01483

**Published:** 2018-10-17

**Authors:** Qiang Li, Xiaoying Chen, Daowei Zhou

**Affiliations:** ^1^Northeast Institute of Geography and Agroecology, Chinese Academy of Sciences, Changchun, China; ^2^Jilin Provincial Key Laboratory of Grassland Farming, Northeast Institute of Geography and Agoecology, Chinese Academy of Sciences, Changchun, China

**Keywords:** grass-legume mixture, N fixation, nutrient concentration, nutrient resorption, litter, soil water availability

## Abstract

The objective was to determine soil properties, and shoot nutrients and nutrient resorption of typical Eurasian steppe species *Leymus chinensis* in various legume mixtures (legume abundance was 0, 25, 50, and 75%). Mixtures with 25 or 50% legume significantly increased soil moisture and soil [N, P] availabilities. Increasing legume abundance enhanced stem and total biomass of green *L. chinensis* shoots, further enhanced the proportion of stem biomass by 14–24% in senesced *L. chinensis* shoots. Legume mixtures, especially 50% legume, enhanced green and senesced organs N concentrations and N pools of *L. chinensis.* Similarly, mixtures with 25 or 50% legume enhanced P concentration and pool of senesced *L. chinensis* shoots, whereas both were decreased by 75% legume. As legume abundance increased, contribution ratios of stem to total N and P pools of senesced *chinensis* shoots increased from 25 to 32 and 25 to 33%, respectively. Mixtures, especially 25 or 50% legume, decreased N and P resorption efficiency (NRE) of *L. chinensis* shoots, whereas 75% legume increased PRE of *L. chinensis*. Total resorbed nutrients may remain stable under varying soil conditions for *L. chinensis*, and resorption of nutrients was symmetric between leaf and stem. In conclusion, legume abundance affected nutrient uptake and return of grass in mixed grasslands, but high legume abundance meant no low N resorption and high litter N content of grass. Furthermore, with increasing legume abundance, stems had more important roles in driving plant production, nutrient utilization, and nutrient return of *L. chinensis.*

## Introduction

Nutrient concentrations in green and senesced plant organs reflect soil fertility and affect organ-level nutrient use efficiency for dry matter production (Güsewell, 2005; Hayes et al., 2014). Nutrient concentrations of senesced organs also strongly influence ecosystem nutrient cycling by altering litter quality and thus litter decomposition (Manzoni et al., 2010; Freschet et al., 2012). Plant nutrients can be withdrawn from senescing organs and subsequently transported into storage organs or into new and growing organs (defined as nutrient resorption; Killingbeck, 1986). Nutrient resorption reduces plant dependence on external nutrient supplies, e.g., soil and fertilizer; this is an internal plant mechanism to adapt to adverse environmental conditions, especially in low-fertility environments (Aerts, 1996; Yuan and Chen, 2009). As an important nutrient conservation strategy, nutrient resorption may contribute to plant nutrient balance and thus ecosystem stability when environment changes (Cleveland et al., 2013; Deng et al., 2016).

Various aspects of plant nutrient content and nutrient resorption have been studied, from molecular to whole-plant levels, among organs and species (Freschet et al., 2010; Lü et al., 2012a; Sun et al., 2018) and in various environments (Hayes et al., 2014; Mayor et al., 2014). Leaves have received the most attention, with much less focus on nutrients and nutrient resorption in other plant organs, including stems (Freschet et al., 2010; Lü et al., 2012a; Sun et al., 2018). Although stems are often nutrient-poor, with less contributions to nutrient uptake and return than leaves, it is noteworthy that stems occupy 37.5–58.2% of above-ground biomass in grasses (Freschet et al., 2010; Lü et al., 2012a). Therefore, stem have important roles in plant-soil feedback (Lü et al., 2012a; Mao et al., 2013).

Soil N availability regulates plant growth and physiology (Vitousek and Howarth, 1991; Harpole et al., 2007), emphasizing its importance for modifying plant nutrients and nutrient resorption (Aerts and Chapin, 2000; Lü and Han, 2010). In general, N enrichment enhances both N and P concentrations in green and senesced leaves (Lü et al., 2013; Yuan and Chen, 2015; Li L. et al., 2016). However, there is uncertainty regarding effects of N availability on plant nutrient resorption efficiencies. In a meta-analysis, Yuan and Chen (2015) concluded that increasing soil N availability tended to decrease leaf N and P resorption efficiencies, with similar results reported from a N fertilization experiment in a steppe grassland (Lü et al., 2013). However, it was suggested that N enrichment likely increased leaf P resorption efficiency depending on plant species (Li L. et al., 2016), with similar results in another N fertilization experiment in a northern grassland of China (Lü and Han, 2010). In contrast, Mayor et al. (2014) suggested that chronic N additions had limited effects on nutrient resorption efficiencies. Based on those inconsistencies, perhaps other factors (e.g., water, plant species, or other nutrients) alter effects of N on nutrient resorption. Significant effects of water and P, and interactions between them and N on plant nutrients and nutrient resorption, have been widely confirmed (Lü and Han, 2010; Mayor et al., 2014).

Legume mixtures are often used to improve soil N fertility in grassland ecosystems, as N fixation and transfer into soil makes legumes an efficient and eco-friendly N source (Li et al., 2015; Peoples et al., 2015; Li Q. et al., 2016). In contrast to use of N fertilizer, legume mixtures have many influences on the environment around plants. For example, legume mixtures can alter inter-specific relationships through direct species introduction. In addition, legume introduction may change soil water availability by altering canopy cover and root distribution and thus water infiltration and utilization (Li et al., 2015; Wu et al., 2016; Yuan et al., 2016). Moreover, legume mixtures likely intensify the P limitation for co-existing plants (Siddique et al., 2008), due to high P demand for legume growth and N_2_ fixation (Augusto et al., 2013), and decreased soil P availability (Crème et al., 2016; Yuan et al., 2016). Therefore, legume mixtures have much more complex effects than direct N fertilization on plant nutrients and nutrient resorption. However, influences of legume mixtures on nutrients and nutrient resorption of associated plants are not well-characterized. Although relative abundance of legumes in mixture communities affect soil properties (Li et al., 2015; Li Q. et al., 2016), effects on plant nutrients and nutrient resorption by various plant organs are not well-characterized.

Objectives of this study were to determine effects of legume abundance on soil water, N input and soil nutrient availability, and their subsequent influences on green and senesced shoot biomass, nutrients and nutrient resorption of a typical Eurasian steppe species. Differences between leaf and stem in nutrients and nutrient resorption were also characterized. We hypothesized that: (a) increasing legume abundance enhances shoot N concentration and reduces shoot N resorption efficiency, due to an increase in N_2_ fixation and soil N availability; (b) increasing legume abundance reduces shoot P concentration and increases shoot P resorption efficiency, due to decreased soil P availability.

## Materials and Methods

### Study Site

The study was conducted at the Changling Grassland Farming Research Station (E123°31′, N44°33′; Elevation 145 m), located at the eastern edge of the Eurasian steppe. At this site, mean annual temperature is 4.9°C and average growing season is ∼150 days. During 2000–2010, annual precipitation was 364 mm at this site (70–80% from June to September). Soil type is meadow chernozem soil. Mature vegetation is meadow steppe dominated by *Leymus chinensis*, a widely distributed perennial C_3_ grass in Eurasian steppe. The current experiment was conducted in a section of meadow that had been converted to cropland and subsequently abandoned. At the start of this experiment, means were: soil pH 8.1, bulk density 1.48 g cm^-3^, organic matter concentration 16 g kg^-1^ and total N concentration 1.1 g kg^-1^.

### Experimental Design

Experimental grasslands were established in 2006, using a completely randomized block design with four replicates. There were four 3 m × 3 m plots in each block. *L. chinensis* monoculture [no legume (N-L)], 75% *L. chinensis* + 25% *Medicago sativa* mixture [low legume abundance (Low-L)], 50% *L. chinensis* + 50% *M. sativa* mixture [medium legume abundance (Mid-L)], and 25% *L. chinensis* + 75% *M. sativa* mixture [high legume abundance (High-L)] were randomly assigned to plots within each block. In detail, in July 2006, *L. chinensis* and *M. sativa* seeds were mixed according to treatments and uniformly sown into plots with 15-cm row spaces. For each plot, total plant density was 600 plant individuals m^-2^, representative of mean plant density in natural meadow communities in this region. Prior to sowing, germination potential of *L. chinensis* and *M. sativa* seeds were assessed and all *M. sativa* seeds were exposed to 98% H_2_SO_4_ for 15 min to break the hard seed coat. No inoculation was applied when legume seeds were sown, as the site had a history of legume presence and in a previous study (Li et al., 2015), soil contained sufficient rhizobia to induce root nodulation. To promote seedling emergence, during the month after sowing, plots were irrigated if there was no rainfall for four consecutive days. In August 2006, all plots were thinned to designated plant densities. To promote establishment of *L. chinensis* and *M. sativa*, other plant species were limited by weeding in 2006 and 2007. After 2007, no weeding was done, as there were limited weeds present, with negligible impact on growth of *L. chinensis* and *M. sativa.* Only initial proportions of plant species were controlled.

### Field Sampling and Analyses

On 2 September, 2010, when grasses were fully matured and before the onset of senescence, a 1 m × 1 m quadrant was designated in the center of each plot for collecting plant samples. Plants within each quadrant were identified according to species, counted and shoot material for each species separately clipped at the soil surface and taken to the laboratory. In each quadrant, 20 mature shoots of *L. chinensis* with similar height were selected, two mature and intact green leaves (without sheath, usually the third and fourth leaves from the top of the shoot) and basal stem (with sheath, 0–5 cm from the bottom of the shoot) were collected on each of these 20 shoots, and then aggregated by organ for measuring biomass and [N, P] concentrations and [N, P] resorption efficiencies. Remaining leaf and stem materials for those 20 shoots of *L. chinensis* were collected to determine biomass and [N, P] concentrations. Within each quadrant, remaining intact *L. chinensis* and *M. sativa* shoots (whole plant with leaf and stem) were retained to determine biomass and N concentration of *M. sativa* shoots and ^15^N isotope of *L. chinensis* and *M. sativa* shoots. Plant samples were oven-dried at 65°C for 48 h and dry weights determined. Plant materials were finely ground and sieved. Total N concentration was determined with a Kjeldahl method (Sparks et al., 1996), total P concentration determined by persulfate oxidation followed by colorimetric analysis (Lü et al., 2013), and ^15^N natural abundance analyzed using a continuous flow Isotope Ratio Mass Spectrometer (Stable Isotope Facility, University of California, Davis, Davis, CA, United States). In mid-October, when plants were completely senesced, a new 1 m × 1 m quadrant was designated and 20 shoots of *L. chinensis* were selected, similar in height to shoots selected on 2 September. Senesced leaf and stem samples were collected and analyzed as described above for green shoots.

In early September, a week after precipitation occurred, three soil samples at a depth of 0–10 cm were collected from each plot, using a soil auger (5-cm diameter). Samples were oven-dried at 105°C for 48 h and gravimetric water content determined. An additional three soil samples were collected (0–10 cm) and passed through a 2-mm sieve to remove plant materials. Soil available N (ammonium + nitrate) concentration was determined using a Bran-Luebbe AA3 autoanalyzer (Bran and Luebbe, Hamburg, Germany) after extracting soil with 50 ml of 2 M KCl. Soil available P concentration was analyzed using the molybdenum blue-ascorbic acid method after extracting soil with 0.5 M NaHCO_3_ (Olsen et al., 1954).

### Calculations

The ^15^N natural abundance method was used to estimate symbiotic N_2_ fixation of legumes. Percentages of N in the legume derived from direct symbiotic N_2_ fixation (%Nsymfix) were estimated using the following formula (Unkovich et al., 2008):

$$\%Nsymfix=\left(\delta^{15}N_{referenceplant}-\delta^{15}N_{legume})/(\delta^{15}N_{referenceplant}-B\right)$$

where δ^15^N is the atom percent excess ^15^N relative to atmospheric N. The subscript ‘legume’ represents *M. sativa*, and the subscript ‘reference plant’ represents *L. chinensis* growing in same plot with legumes. *L. chinensis* was used as the only reference plant, as estimated % Nsymfix of *M. sativa* using *L. chinensis* as a reference plant could represent the mean estimate using four different reference plants (Li et al., 2015). The B-value was based on our previous study (Li et al., 2015). Total Nsymfix was estimated based on %Nsymfix, legume shoot biomass and shoot N concentration.

Nutrient pools of green and senesced leaf and stem were calculated based on their respective mass and nutrient concentrations. Nutrient resorption efficiency (NuRE) was estimated based on nutrient pools of green and senesced leaf and stem, calculated as:

$${NuRE}_{pool}=(1-{Nutrientpool}_{senesced}/{Nutrientpool}_{green})\times 100\%$$

where Nutrient pool_senesced_ and Nutrient pool_green_ were the N or P pool of senesced and green organs (leaf or stem) in each plot, respectively.

Amounts of nutrients resorbed from organs (leaves and stem) were calculated based on the product of nutrient pools NuRE of green organs. Amounts of nutrients in senesced organs were calculated as product of weights and nutrient concentrations of senesced organs (Lü et al., 2012a).

### Data Analyses

Two-way ANOVA was used to determine main and interactive effects of block and legume abundance on soil properties, symbiotic N fixation, and biomass, nutrients, and nutrient resorption for each shoot tissue. Based on preliminary analyses, effects of block and interactive effects of block and legume abundance on these measured variables were not significant (*P* > 0.05). Paired Student’s *t*-tests were used to detect differences between leaf and stem for biomass, nutrients and nutrient resorption. Simple linear regressions were performed to analyze relationships between soil water content and %Nsymfix of *M. sativa*. The control power of soil water content, soil [N, P] availability and soil available N:P to organ biomass, nutrient concentrations, and nutrient resorption efficiency were examined by multiple stepwise regressions. Means were compared with a Duncan’s test. Significance for all statistical tests was defined as *P* = 0.05. All data were analyzed using SPSS17.0 software (Chicago, IL, United States).

## Results

At 4 years after planting, the combination of *L. chinensis* and *M. sativa* occupied 93 and 94% of total plant density and aboveground biomass, respectively, of the entire sward. Relative densities of *M. sativa* and *L. chinensis* were 0:1, 0.34:1, 0.93:1, and 2.83:1 for NL, Low-L, Mid-L and High-L treatments, respectively, similar to relative density at sowing.

**FIGURE 1:**
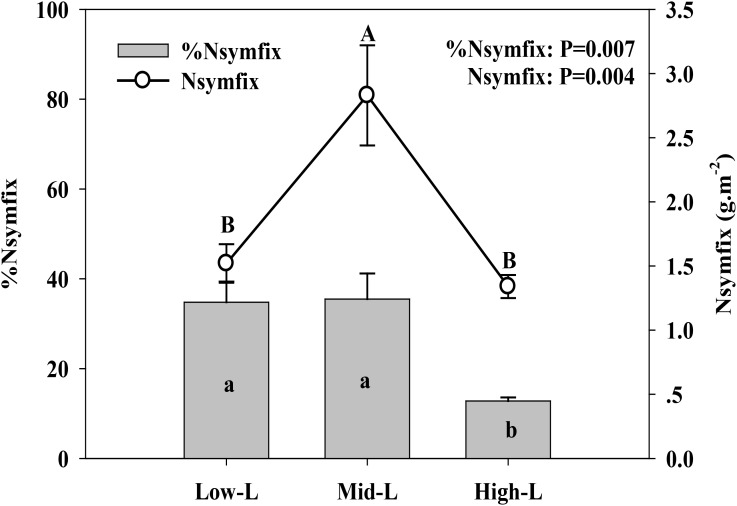
Mean ± SEM (*n* = 4) %Nsymfix and Nsymfix of *Medicago sativa* under mixtures with varying legume abundance. Low-L, mixture with 75% *L. chinensis* + 25% *M. sativa*; Mid-L, mixture with 50% *L. chinensis* + 50% *M. sativa*; High-L, mixture with 25% *L. chinensis* + 75% *M. sativa.*^a,b^%Means without a common superscript differed (*P* < 0.05). ^A,B^Means without a common superscript differed (*P* < 0.05).

**FIGURE 2:**
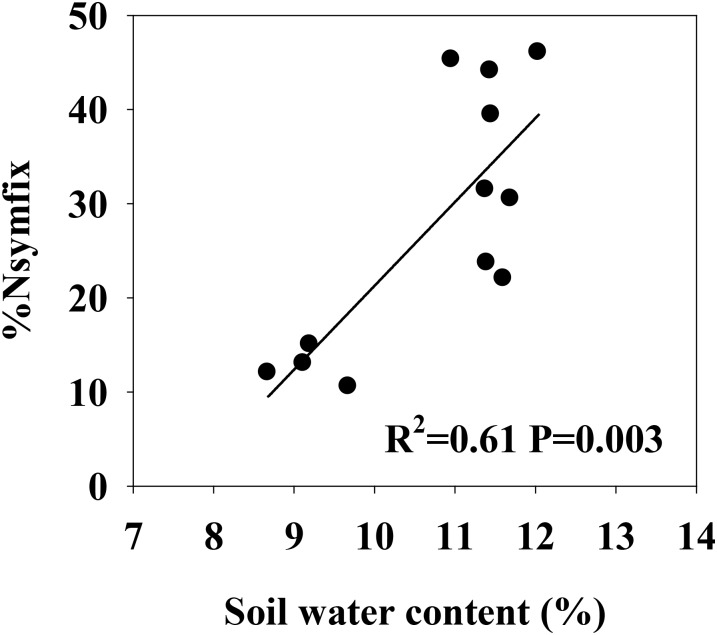
Linear relationship between soil water content (0–10 cm) and %Nsymfix of *M. sativa*.

Soil moisture at 0–10 cm was significantly influenced by legume mixture, with higher values for Low-L and Mid-L compared to N-L and High-L treatments (*P* < 0.001, **Table 1**). Low-L and Mid-L treatments increased soil inorganic N and available P concentration compared to N-L and High-L treatments (*P* < 0.01, **Table 1**). In general, as legume abundance increased, soil available N:P was enhanced (*P* < 0.001, **Table 1**). High-L treatment significantly decreased %Nsymfix in *M. sativa*, whereas *M. sativa* fixed more atmospheric N into grassland under Mid-L compared to other treatments (**Figure 1**). Soil water content was positively related to %Nsymfix in *M. sativa* (**Figure 2**).

**Table 1:** Mean ± SEM (*n* = 4) soil moisture and nutrient characteristics under varying legume abundances.

Treatments	Soil water content (%)	Soil inorganic N concentration (mg kg^-1^)	Soil available P concentration (mg kg^-1^)	Soil available N:P
N-L	9.29 ± 0.31^b^	22.09 ± 0.33^c^	2.89 ± 0.10^b^	7.66 ± 0.24^b^
Low-L	11.31 ± 0.11^a^	27.84 ± 0.23^b^	3.49 ± 0.07^a^	7.99 ± 0.09^b^
Mid-L	11.70 ± 0.13^a^	32.80 ± 0.76^a^	3.22 ± 0.09^a^	10.20 ± 0.33^a^
High-L	9.17 ± 0.20^b^	27.77 ± 0.33^b^	2.79 ± 0.11^b^	10.00 ± 0.34^a^


*N-L, no legume; Low-L, 25% legume; Mid-L, 50% legume; High-L, 75% legume.*

*^*a,b,c*^Within a soil trait, means without a common superscript differed (*P* < 0.05).*

**FIGURE 3:**
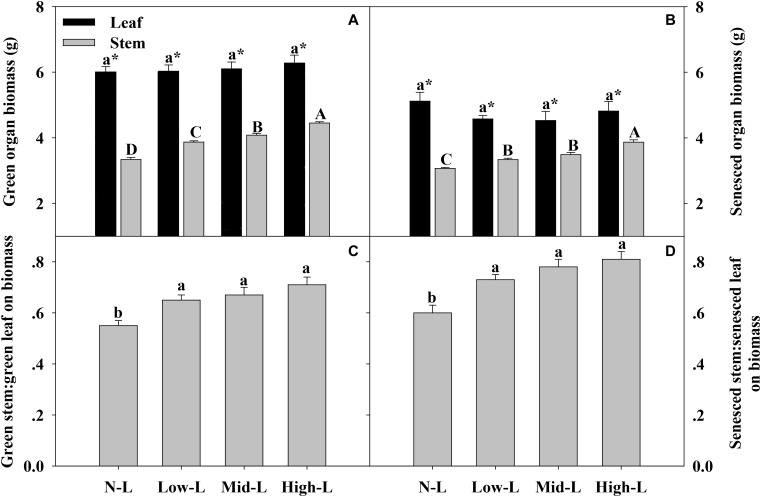
Green organ biomass **(A)** and senesced organ biomass **(B)**, green stem biomass: green leaf biomass **(C)** and senesced stem biomass: senesced leaf biomass **(D)** for 20 *L. chinensis* shoots under monocultures and mixtures with varying legume abundance. Error bars indicate + 1 SE (*n* = 4). N-L, *L. chinensis* monoculture; Low-L, mixture with 75% *L. chinensis* + 25% *M. sativa*; Mid-L, mixture with 50% *L. chinensis* + 50% *M. sativa*; High-L, mixture with 25% *L. chinensis* + 75% *M. sativa*. ^a,b^Means without a common superscript differed (*P* < 0.05). ^A,B,C^ Means without a common superscript differed (*P* < 0.05). ^∗^Within a legume mixture, difference between leaf and stem (*P* < 0.05).

**FIGURE 4:**
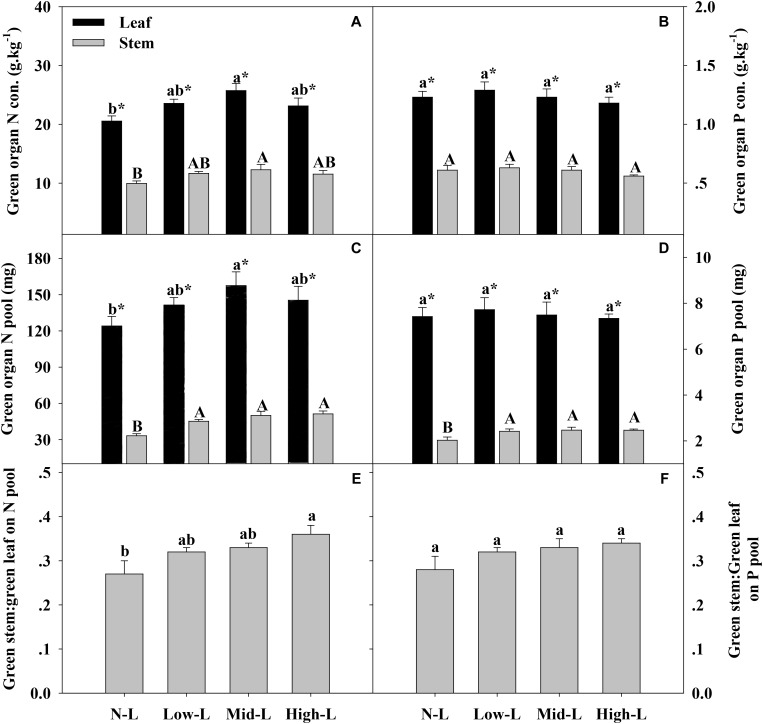
Green organ N concentration **(A)**, green organ P concentration of *L. chinensi*s **(B)**, green organ N pool **(C)**, green organ P pool **(D)**, green stem N pool: green leaf N pool **(E)** and green stem P pool: green leaf P pool **(F)** for 20 *L. chinensis* shoots under monocultures and mixtures with varying legume abundance. Error bars indicate + 1 SE (*n* = 4). N-L, *L. chinensis* monoculture; Low-L, mixture with 75% *L. chinensis* + 25% *M. sativa*; Mid-L, mixture with 50% *L. chinensis* + 50% *M. sativa*; High-L, mixture with 25% *L. chinensis* + 75% *M. sativa*. ^a,b^Means without a common superscript differed (*P* < 0.05). ^A,B,C^Means without a common superscript differed (*P* < 0.05). ^∗^Within a legume mixture, difference between leaf and stem (*P* < 0.05).

**FIGURE 5:**
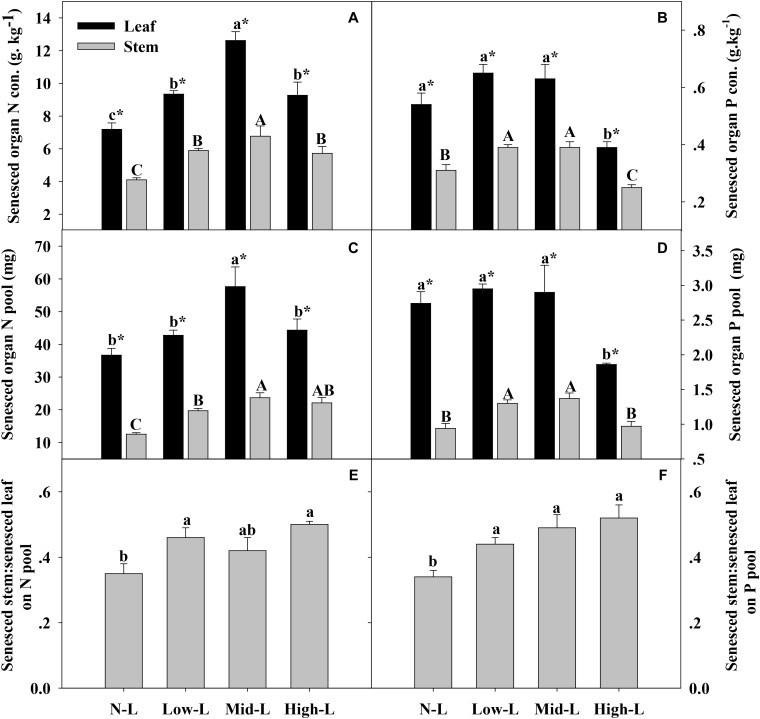
Senesced organ N concentration **(A)**, senesced organ P concentration **(B)** senesced organ N pool **(C)**, senesced organ P pool **(D)**, green stem N pool: green leaf N pool **(E)** and green stem P pool: green leaf P pool **(F)** for 20 *L. chinensis* shoots under monocultures and mixtures with varying legume abundance. Error bars indicate + 1 SE (*n* = 4). N-L, *L. chinensis* monoculture; Low-L, mixture with 75% *L. chinensis* + 25% *M. sativa*; Mid-L, mixture with 50% *L. chinensis* + 50% *M. sativa*; High-L, mixture with 25% *L. chinensis* + 75% *M. sativa*. ^a,b^Means without a common superscript differed (*P* < 0.05). ^A,B,C^Means without a common superscript differed (*P* < 0.05). ^∗^Within a legume mixture, difference between leaf and stem (*P* < 0.05).

Regardless of legume abundance, leaf of *L. chinensis* had significantly higher biomass and nutrient concentrations compared to stem, in both green and senesced states (**Figures 3**–**5**). Increasing legume abundance enhanced both green and senesced stem biomass of *chinensis* shoots (*P* < 0.01), but did not significantly alter green or senesced leaf biomass of *chinensis* shoots (*P* > 0.05); therefore, the stem biomass: leaf biomass ratio increased 18–29 and 22–35% in green and senesced shoots, respectively (*P* < 0.05, **Figures 3C,D**).

*Medicago sativa* mixtures, especially Mid-L, increased N concentration of green leaf and stem of *L. chinensis* compared to NL treatments (*P* < 0.05, **Figure 4A**). However, legume mixture did not alter P concentrations of green leaf and stem of *L. chinensis* (*P* > 0.05, **Figures 4A,B**). Legume mixture enhanced N stock in green leaf and stem, and P stock in green stem of *L. chinensis* (*P* < 0.05), but had not significant effect on P stock of green leaf (*P* > 0.05). Across all treatments, *L. chinensis* stem stored 21–26% of N pool and 22–25% of P pool in green shoot (**Figures 4E,F**). *M. sativa* mixtures, especially Mid-L, increased N concentrations and N stock of senesced leaf and stem of *L. chinensis* compared to NL treatments (*P* < 0.05, **Figures 5A,C**). Similarly, Low-L and Mid-L treatments increased P concentrations and P stock of senesced leaf and stem of *L. chinensis.* However, the High-L treatment significantly decreased P concentration of senesced leaf and stem, and P stock of senesced leaf for *L. chinensis* compared to N-L treatment (**Figures 5B,D**). The stem:leaf ratio for N and P pool increased 15–28 and 19–30% in senesced shoots following legume mixture, respectively (*P* < 0.05, **Figures 5E,F**).

**FIGURE 6:**
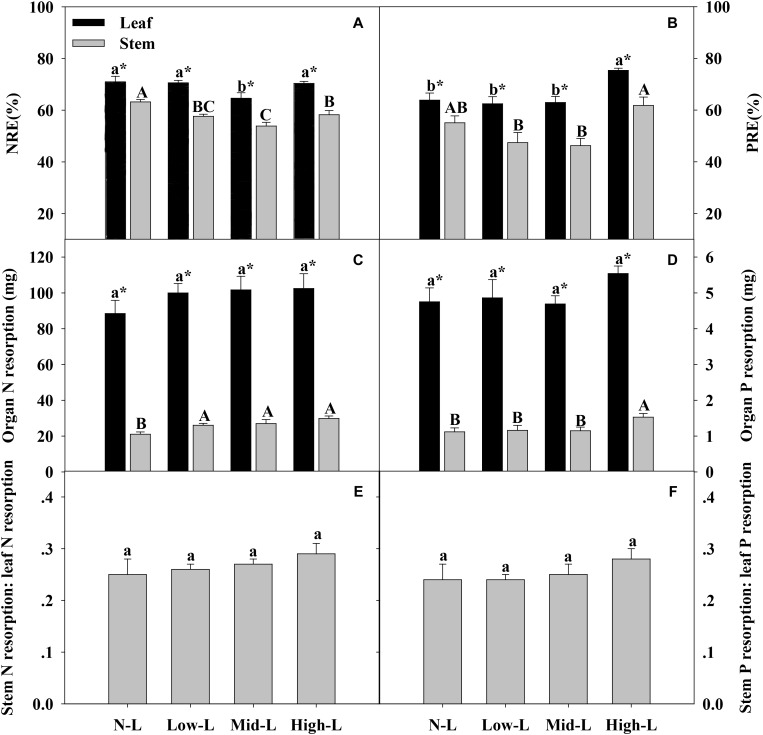
Organ N resorption efficiency (NRE, **A**), organ P resorption efficiency (PRE, **B**), amount of organ N resorption **(C)**, amount of organ P resorption **(D)**, stem N resorption: leaf N resorption **(E)** and stem P resorption: leaf P resorption **(F)** for 20 *L. chinensis* shoots under monocultures and mixtures with varying legume abundance. Error bars indicate + 1 SE (*n* = 4). N-L, *L. chinensis* monoculture; Low-L, mixture with 75% *L. chinensis* + 25% *M. sativa*; Mid-L, mixture with 50% *L. chinensis* + 50% *M. sativa*; High-L, mixture with 25% *L. chinensis* + 75% *M. sativa*. ^a,b^Means without a common superscript differed (*P* < 0.05). ^A,B,C^Means without a common superscript differed (*P* < 0.05). ^∗^Within a legume mixture, difference between leaf and stem (*P* < 0.05).

Only Mid-L treatment significantly decreased leaf N resorption efficiency (NRE) of *L. chinensis* compared to NL treatment. All mixtures, especially Mid-L, decreased stem N resorption efficiency (NRE) of *L. chinensis* compared to N-L treatment (*P* < 0.05, **Figure 6A**). High-L treatment significantly increased leaf P resorption efficiency (PRE) of *L. chinensis* compared to the other three treatments. The stem PRE of *L. chinensis* was significantly higher under High-L treatment compared to Low-L and Mid-L treatments, with no significant difference from the N-L treatment (**Figure 6B**). Increased legume significantly increased N resorption from stem (*P* < 0.05), but had no significant influence on N and P resorption from leaf of *L. chinensis* (*P* > 0.05). High-L treatment significantly enhanced P resorption from stem of *L. chinensis* (**Figures 6C,D**). Ratio of N and P resorbed from stem relative to leaf remained stable under varied legume-grass mixtures, ranging from 0.25–0.29 to 0.24–0.28, respectively (*P* > 0.05, **Figures 6E,F**).

For *L. chinensis*, the biomass of green and senesced stem and N concentrations of green and senesced organs principally were positively related to soil inorganic N concentrations (**Table 2**), P concentrations of senesced leaf and stem principally were positively related to soil water content, soil available N:P had principal control of leaf NRE, whereas soil inorganic N concentration had principal control of stem NRE, and stem PRE primarily had a negative association with soil water content.

**Table 2:** Dependence (multiple regressions) of tissue biomass, tissue nutrients concentration, and tissue nutrient resportion efficiency (NRE, PRE) on various aspects of soil, for green and senesced *L. chinensis* shoots.

	Response variable	Regression parameters for each dependent variable						
		
		Intercept	Soil water content (%)	Soil inorganic N concentration (mg.kg^-1^)	Soil available P concentration (mg.kg^-1^)	Soil available N:P	Overall R^2^	Overall *F*-value
Leaf	Green leaf biomass (g) NNrecruited species	20.060	-0.166	0.502	-3.824	-1.582	0.372	1.629
	Green leaf N concentration (g.kg^-1^)	10.082^∗^	0.090	0.477^∗∗^	0.259	-0.353	0.501	14.065^∗∗^
	Green leaf P concentration (g.kg^-1^) (g m^-2^)	1.064	-0.023	-0.002	0.149	0.007	0.056	0.163
	Senesced leaf biomass (g)	6.493	-0.171	-0.188	-0.193	-0.238	0.318	6.527
	Senesced leaf N concentration (g.kg^-1^)	-4.066^∗^	-0.045	0.495^∗∗∗^	-0.083	0.065	0.793	53.601^∗∗∗^
	Senesced leaf P concentration (g.kg^-1^)	-0.490	0.079^∗^	-0.244	0.302	-0.219	0.378	8.503^∗^
	NRE (%)	86.487^∗∗∗^	-0.281	-0.321	-0.181	-1.936^∗^	0.382	8.654^∗^
	PRE (%)	22.234	-3.888	-2.62	24.878	10.06	0.377	1.663
Stem	Green stem biomass (g) NNrecruited species	2.327^∗∗∗^	-0.376	0.045^∗∗∗^	-0.241	0.368	0.594	20.472^∗∗∗^
	Green stem N concentration (g.kg^-1^)	5.248^∗^	-0.062	0.221^∗∗^	0.196	-0.254	0.392	9.040^∗∗^
	Green stem P concentration (g.kg^-1^) (g m^-2^)	0.251	0.035	-0.009	0.006	0.012	0.105	0.857
	Senesced stem biomass (g)	2.482^∗∗∗^	-0.440	0.020^∗^	-0.105	0.153	0.348	7.463^∗^
	Senesced stem N concentration (g.kg^-1^) (g.kg^-1^)	-0.934	0.027	0.237^∗∗∗^	0.116	-0.129	0.725	36.845^∗∗∗^
	Senesced stem P concentration (g.kg^-1^)	-0.391^∗^	0.055^∗∗∗^	-0.217	0.138	-0.164	0.592	20.310^∗∗∗^
	NRE (%)	79.929^∗∗∗^	-0.134	-0.785^∗∗^	-0.078	0.075	0.589	20.029^∗∗^
	PRE (%)	133.813^∗∗^	-6.198^∗∗^	0.282	-0.360	0.266	0.471	12.459^∗∗^


*^∗^*P* < 0.05, ^∗∗^*P* < 0.01, ^∗∗∗^*P* < 0.001.*

## Discussion

### N_2_ Fixation, Soil N and P Availability Were Affected by Legume Abundance

It was expected that increasing legume abundance would enhance soil N availability by increasing symbiotic N_2_ fixation. Symbiotic N_2_ fixation was determined by %Nsymfix in legume and legume biomass. In this study, %Nsymfix of legume remained stable as sowing proportion of legume increased from 25 to 50%, although an increased legume biomass resulted in increasing symbiotic N_2_ fixation. However, high legume abundance likely intensified intra-specific competition of *M. sativa* and reduced stimulation of grass to N_2_ fixation of *M. sativa* (Ledgard and Steele, 1992). Moreover, legume %Nsymfix can be greatly limited by soil water content (Austin et al., 2004; Li et al., 2015). *M. sativa* had more developed canopy and root system compared with *L. chinensis.* Therefore, *M. sativa* substitution to *L. chinensis* will increase the shoot and root biomass of entire sward (Li et al., 2015). In this study, low-middle legume abundance may have improved soil water content through increasing rainfall infiltration, due to a increased root biomass (Li et al., 2015; Wu et al., 2016), promoting symbiotic N_2_ fixation. Further increased legume abundance may have not induced more positive effect on rainfall infiltration, but can induce more water transpiration due to increased biomass and leaf area of plant canopy (Li et al., 2015), thereby decreasing soil water content under the highest legume abundance. This could cause a physiological limitation of N_2_ fixation (Austin et al., 2004; Li et al., 2015) and induce a physiological transformation from fixing atmospheric N to capturing soil N for *M. sativa* (Li Q. et al., 2016). Consequently, %Nsymfix in *M. sativa* sharply declined as relative legume abundance increased from 50 to 75%, with a 53% decrease in induced symbiotic N_2_ fixation and changes in soil inorganic N concentrations.

Effects of legume mixtures on soil available P remain controversial. In some studies, legume mixture reduced soil available P concentration (Crème et al., 2016; Yuan et al., 2016). In contrast, others concluded that legume intercrops facilitated rhizosphere P mobilization, which may increase available P concentrations in soil (Li et al., 2007, 2014). Based on the present results, we inferred that effects of legume mixture on soil available P depended on legume abundance. Further, based on linear regression analysis, soil moisture had a positive association with soil P availability (*R*^2^ = 0.66, *P* = 0.001). Lower soil moistures under N-L and high-L treatments likely improved soil redox potential, more iron oxide and hydroxide minerals bound with P, removing P from the soil exchange complex and significantly decreasing P availability compared to Low-L and Mid-L treatments (Wood and Silver, 2012).

### Stem Was an Important Component to Drive Nutrient Utilization and Return of *L. chinensis*

As a support organ, stem is often nutrient-poor and decomposes slowly after senescence. Therefore, stem has received much less attention than leaf regarding plant nutrition, nutrient utilization and nutrient return *via* decomposition (Freschet et al., 2010; Lü et al., 2012a; Sun et al., 2018). Regardless, stem is an important component of above-ground biomass in grass (Freschet et al., 2010; Lü et al., 2012a). Compared to leaf, the importance of stem in driving nutrient utilization and return of grass is usually underestimated. In the current study, stem comprised >36% of green and senesced *L. chinensis* shoots biomass, stored >21% of N and P pool in green *L. chinensis* shoot and contributed >25% of N and P pool in senesced *L. chinensis* shoot. Moreover, resorbed N and P from stem accounted for >19% of total resorbed N and P pools from *L. chinensis* shoot. Stem is an important plant component involved in nutrient uptake, nutrient resorption and nutrient return of *L. chinensis* (Freschet et al., 2010; Lü et al., 2012b). In addition, increasing legume abundance enhanced canopy cover, which may intensify light competition between neighboring plants (Lane et al., 2000). Increased height of *L. chinensis* to acquire more light (mean plant height increased from 35 to 43 cm as legume abundance increased from 0 to 75%) increased stem biomass and thus proportion of stem in whole *L. chinensis* shoot biomass and nutrient pools. Consequently, stem has increasing importance to drive nutrient utilization and nutrient return of *L. chinensis* as legume abundance increases in a mixed grassland.

### Responses of Nutrients and Nutrient Resportion of Leaf and Stem to Varying Legume Mixtures

Altered legume abundance drives changes in soil N availability, which is expected to have important influences on plant growth and nutrient uptake (Nyfeler et al., 2011; Li Q. et al., 2016). However, legume abundance had differential effects on N uptake of green stem and green leaf of *L. chinensis*. Legume abundance primarily changed N uptake of *L. chinensis* leaf by influencing leaf N concentration. As a consequence, the N pool of green leaf of *L. chinensis* was highest in Mid-L, due to highest soil N availability. By contrast, legume abundance primarily changed N uptake of green stem by influencing stem biomass. Consequently, there was a continuous increase of green stem N pool as legume abundance increased, although high legume abundance decreased soil N availability. Furthermore, increasing light competition may have intensified N uptake of stem of *L. chinensis* by stimulating stem growth. Overall, N pools in green shoots were enhanced by 19–32% in legume mixtures, peaking in mixtures with 50% legume. Contrary to expectations, P concentration of green leaf and stem of *L. chinensis* remained stable, despite legume abundance changing soil water and [N, P] availabilities. Similar results were reported in water and N amendment experiments conducted in a steppe grassland (Lü and Han, 2010; Huang et al., 2018). Although legume introduction significantly increased the P pool of green stem, the total P pool in green leaf and whole shoots of *L. chinensis* less were changed by legume mixture. Therefore, we inferred that leaf and total P uptake of *L. chinensis* we independent of changes in soil P availability driven by legume content.

Compared to green organs, [N, P] concentrations of senesced organs were more clearly regulated by legume-driven environmental changes (**Figure 5** and **Table 2**). Positive associations between soil N availability and N concentrations of senesced organs were attributed to positive plant-soil feedback through litter decomposition (Lü et al., 2012a; Wang et al., 2017). With more N uptake in green organs, senesced organs of *L. chinensis* reserved more N pools in mixtures with 50% legume than in other treatments. However, N pools of senesced leaf and stem had asymmetric changes as legume abundance changed. We concluded that increased legume abundance significantly increased senesced stem biomass, but did not affect senesced leaf biomass. Consequently, as legume abundance increased from 0 to 75%, contributions of stem to total N pool of senesced *L. chinensis* shoot increased from 25 to 32%. Soil water content, rather than soil P availability, had primary associations with P concentrations of senesced organs for *L. chinensis*, emphasizing the importance of soil moisture in modifying litter P concentrations in this semi-arid grassland. However, in another study with similar climate, soil water had no significant effect on P concentration of senesced leaf (Huang et al., 2018). Similar results were reported by Lü and Han (2010). To our knowledge, less information is available regarding effects of soil moisture on litter P concentration. In our study, soil moisture was positively related to soil available P concentrations. Perhaps changes in soil water affects soil P availability, altering P concentrations in senesced organs. The High-L treatment decreased leaf and total P pools of senesced *L. chinensis* shoot compared to N-L treatment, due to decreased P concentrations of senesced organs. Similar to the N pool, the contribution of stem to total P pool of senesced *L. chinensis* shoot increased from 25 to 33% as legume abundance increased from 0 to 75%. Based on significant effects of legume abundance on nutrient concentrations and biomass components of senesced *L. chinensis* shoots, we inferred that legume mixtures may strongly influence decomposition and nutrient return of *L. chinensis* litter. For example, with low and mid legume abundance, increased organ nutrient concentrations and nutrient pools may accelerate decomposition of *L. chinensis* litter and increase nutrient release into soil (Manzoni et al., 2010; Freschet et al., 2012). However, as legume abundance increased, a greater proportion of stem to whole litter mass may delay complete decomposition of *L. chinensis* litter, as decomposition of stem is usually slower than leaf (Manzoni et al., 2010).

For *L. chinensis*, the leaf withdrew 65–71% of N and 63–75% of P during senescence. By contrast, stem had lower resorption efficiency for both N (54–63%) and P (46–62%) (Lü et al., 2012a; Vergutz et al., 2012; Brant and Chen, 2015). Intraspecific organ differences in nutrient resportion may be due to differences between leaf and stem in nutrient status (Kobe et al., 2005), as nutrient resorption efficiency tends to decrease with increased organ nutrient status (Vergutz et al., 2012). Overall, *M. stavia* mixture, especially middle legume abundance, decreased N resorption efficiency of *L. chinensis* shoot, as N_2_ fixation increased soil N availability and soil available N:P (**Table 2**). However, high legume abundance enhanced P resorption efficiency of *L. chinensis*, primarily due to reduced soil water content. Similarly, Lü and Han (2010) reported that a forb species significantly increased leaf P resorption efficiency under water enrichment. In the current study, varying legume mixtures did not significantly change total amount of resorbed nutrients and relative proportions from leaf and stem. Therefore, we inferred that total resorbed nutrients for *L. chinensis* may remain stable under varying soil conditions and nutrient resportion was symmetric in leaf and stem. Consequently, external nutrient content may regulate nutrient uptakes for new and growing organs of *L. chinensis* under variations in plant mixtures and soil fertility.

## Conclusion

Legume abundance affected growth, nutrient uptake and resorption of grass organs in mixed grasslands, by changing the local environment, including soil water and [N,P] availability. Responses and response mechanisms of nutrient uptake and resorption for leaf and stem to legume abundance differ. We concluded that higher legume abundance enhanced stem N uptake and stem litter N pool of *L. chinensis* by promoting stem growth, whereas medium legume abundance promoted leaf N uptake and leaf litter N reserve of *L. chinensis* due to greater soil N availability. Legume abundance had little influence on P uptake of leaf and stem of *L. chinensis*. Regardless, higher soil water may promote P reserves of leaf and stem litter of *L. chinensis*under mixtures with Low to Mid legume abundance. Due to positive effects of increasing legume abundance on stem biomass, it was clear that stem had an important role in driving nutrient utilization and return of *L. chinensis*. Middle legume abundance decreased N resorption efficiency of *L. chinensis* shoot, attributed to high soil N availability and soil available N:P, whereas high legume abundance enhanced shoot P resorption efficiency of *L. chinensis* due to low soil water content. Total resorbed nutrients may remain stable under varying soil conditions for *L. chinensis*, whereas nutrient resportion was symmetric between leaf and stem.

## Author Contributions

QL and XC made equal contributions to this manuscript. QL was responsible for conception, field sampling, and manuscript writing. XC was responsible for sample analyses and manuscript revision. DZ was responsible for experimental design.

## Conflict of Interest Statement

The authors declare that the research was conducted in the absence of any commercial or financial relationships that could be construed as a potential conflict of interest.
